# Pain quality patterns in delayed onset muscle soreness of the lower back suggest sensitization of fascia rather than muscle afferents: a secondary analysis study

**DOI:** 10.1007/s00424-023-02896-8

**Published:** 2023-12-16

**Authors:** Andreas Brandl, Jan Wilke, Christoph Egner, Tobias Schmidt, Andreas Schilder, Robert Schleip

**Affiliations:** 1 Department of Sports Medicine, Institute for Human Movement Science, Faculty for Psychology and Human Movement Science, University of Hamburg, 20148 Hamburg, Germany; 2 Conservative and Rehabilitative Orthopedics, Department of Sport and Health Sciences, Technical University of Munich, 80992 Munich, Germany; 3 Vienna School of Osteopathy, 1130 Vienna, Austria; 4 Department of Movement Sciences, University of Klagenfurt, 9020 Klagenfurt, Austria; 5 Department for Medical Professions, Diploma Hochschule, 37242 Bad Sooden-Allendorf, Germany; 6 Osteopathic Research Institute, Osteopathie Schule Deutschland, 22297 Hamburg, Germany; 7 Institute of Interdisciplinary Exercise Science and Sports Medicine, MSH Medical School Hamburg, 20457 Hamburg, Germany; 8 Department of Orthopaedics and Trauma Surgery, Medical Faculty Mannheim, Heidelberg University, 68167 Mannheim, Germany

**Keywords:** Delayed onset muscle soreness, Eccentric exercise, Pain quality, Pain perception, Muscle pain, Fascia pain, Thoracolumbar fascia, Lumbar multifidus muscle

## Abstract

Delayed onset muscle soreness (DOMS) of the lower back is considered a surrogate for acute low back pain (aLBP) in experimental studies. Of note, it is often unquestioningly assumed to be muscle pain. To date, there has not been a study analyzing lumbar DOMS in terms of its pain origin, which was the aim of this study. Sixteen healthy individuals (L-DOMS) were enrolled for the present study and matched to participants from a previous study (*n* = 16, L-PAIN) who had undergone selective electrical stimulation of the thoracolumbar fascia and the multifidus muscle. DOMS was induced in the lower back of the L-DOMS group using eccentric trunk extensions performed until exhaustion. On subsequent days, pain on palpation (100-mm analogue scale), pressure pain threshold (PPT), and the Pain Sensation Scale (SES) were used to examine the sensory characteristics of DOMS. Pain on palpation showed a significant increase 24 and 48 h after eccentric training, whereas PPT was not affected (*p* > 0.05). Factor analysis of L-DOMS and L-PAIN sensory descriptors (SES) yielded a stable three-factor solution distinguishing superficial thermal (“heat pain “) from superficial mechanical pain (“sharp pain”) and “deep pain.” “Heat pain “ and “deep pain” in L-DOMS were almost identical to sensory descriptors from electrical stimulation of fascial tissue (L-PAIN, all *p* > 0.679) but significantly different from muscle pain (all *p* < 0.029). The differences in sensory description patterns as well as in PPT and self-reported DOMS for palpation pain scores suggest that DOMS has a fascial rather than a muscular origin.

**Supplementary Information:**

The online version contains supplementary material available at 10.1007/s00424-023-02896-8.

## Introduction

Experimentally induced muscle pain is frequently used in research as a model for acute low back pain (aLBP) [3, 10, 31, 35, 55]. As an example, delayed onset muscle soreness (DOMS) provoked by fatiguing trunk extensions serves as a surrogate for aLBP in experimental studies and is expected to provide additional insight into dynamic changes in pain sensitivity due to its gradual offset over several days [35]. It influences nociceptive mechanisms quantitatively assessed by temporal pain summation or conditioned pain modulation, pain sensitivity [35], pain resilience [55], and trunk muscle activity [31]. In a further note, DOMS and fatigue were reported to reduce both trunk repositioning sensation and lumbar spine stability in healthy participants [5, 43]. Thus, many authors contend that DOMS is an equivalent for muscle pain and that it is a general mechanism for creating a standardized painful muscle state [3, 31, 35, 55].

Interestingly, there is neither sufficient evidence to support the traditional hypotheses of DOMS resulting from sarcomere damage [21], lactate production [24], or increase in free radicals [13] nor that pain itself is caused by nociceptive afferents originating from the muscle [39, 57, 58, 65]. In addition, recent studies have shown that the extramuscular connective tissue (ECT), known as the deep fascia, is likely involved in DOMS, showing increased stiffness and thickness [23, 32, 57, 65]. The ECT is characterized by a close mechanical relationship with the adjacent muscle. However, the fascia is not just a packaging organ. The thoracolumbar fascia (TLF) is highly innervated, and most of the afferent fibers appear to have a nociceptive, proprioceptive, or autonomic regulatory function [38, 54]. Even original injuries of the muscle are not exclusively limited to the muscle tissue. Approximately 90% of cases involve the intrinsic site of injury in either the musculotendinous junction or the extramuscular fascia [62]. Like DOMS, muscle injuries often occur after eccentric contractions. Consequently, both may cause similar connective tissue involvement, for which there is compelling evidence for the existence of structural damage to the extracellular matrix in DOMS [9, 46, 57]. Therefore, it is suggested that the TLF is likely involved in the development of aLBP [26, 56, 64].

Verbal pain descriptors such as the Pain Perception Scale “Schmerzempfindungsskala” (SES) in German language [22] are reliable parameters to characterize aLBP. They have been used to assess the processing of pain stimuli [18] and to distinguish pain qualities of muscle and fascia tissue [51]. Multiple descriptors have been used to distinguish, for example, between primary and secondary chronic pain syndromes [60], trigeminal neuralgia and atypical facial pain [37], nociceptive and neuropathic pain [20], and A-delta-mediated and C-fiber-mediated pain [28]. Furthermore, verbal descriptors were used to identify neuropathic components of low back pain [1], optimize pain stimulus processing [18], and examine patients’ sensitivity to words [4]. Schilder and colleagues [51] demonstrated different factorial patterns of electrically stimulated muscle, fascia, and skin in a previous study. Fascia descriptors were found to be very similar to those of skin, including the terms “burning,” “scalding,” and “hot.” In contrast, descriptors for muscle pain were significantly different, e.g., including the label “deep pain” [24]. Models of experimentally induced soft tissue pain are important to overcome the limitations of clinical trials to control the pain experience (e.g., induction in a specific tissue; defined level of the stimulus) of participants [3]. As outlined, DOMS to induce aLBP in particular is often assumed to damage muscles and subsequently cause pain originating in the fascia or muscle [3, 21, 24]. Especially a model such as DOMS, which is commonly used and assumed to mimic muscle pain, needs to be reviewed in the light of new findings in order to determine whether it really holds up to this claim. Considering the new findings on the involvement of the ECT in DOMS [23, 32, 57, 65], the present study investigated the hypothesis that the quality of DOMS pain is related to the deep fascia rather than the muscle. Therefore, we investigated the influence of a maximal eccentric trunk extension exercise protocol to subjective exhaustion, inducing DOMS, on pain descriptors related to fascia and muscle pain.

## Methods

This study has a secondary analysis matched pair design. The first group was obtained from a previous study by Schilder and colleagues [51] investigating pain qualities in the lower back upon selective nociceptive muscle and fascia stimulation (L-PAIN, *n* = 16). The second group, which was matched one-to-one to L-PAIN, was assessed for pain qualities following strenuous trunk extension exercise leading to DOMS in the lower back (L-DOMS, *n* = 16).

The study was prospectively registered with the German Clinical Trials Register (DRKS00031201). It adhered to the STROBE Statement as well as the declaration of Helsinki [66] and was approved by the ethical committee of the Diploma Hochschule, Germany (Nr.1065/2023). All participants provided written informed consent.

### Participants

As L-PAIN with data from the study by Schilder et al. [51] had a sample size of *n* = 16 and was a matched pair trial, we recruited an additional *n* = 16 participants for L-DOMS (total sample size *n* = 32). The primary endpoint was the results of the factor analysis. A power calculation of the factor analysis of the previous study (L-PAIN) [51], collapsed to a three-factor model (“heat pain,” “sharp pain,” “deep pain”; Cohen’s *f* = 0.564, *α* err = 0.05) resulted in a power (1 − *ß*) of 0.93.

Recruitment of L-DOMS was performed via direct contact, a notice board, and the distribution of information material in a school for health professions. Inclusion criteria were generally healthy constitution; body mass index (BMI) between 18 and 29.9; female or male participants aged 18 to 32 years. These inclusion criteria were chosen because it is known from previous studies that fascial tissue changes in morphology, stiffness, and blood flow with increasing age and BMI [11, 15, 52, 53, 61, 63]. Exclusion criteria were generally valid contraindications to exhausting trunk extension exercises (i.e., fractures, tumors, infections, severe cardiovascular, neural, and metabolic diseases); pregnancy; rheumatic diseases; taking medication that affects blood circulation, pain or mind; taking muscle relaxants; skin changes (e.g., neurodermatitis, psoriasis, urticaria, decubitus ulcers, hematoma); overuse disorders, surgery or other scars in the lumbar region; previous mental illness; surgery in the last three months; acute inflammation. The exclusion criteria for participants of the L-PAIN group were any medication, history of chronic pain, or recent surgeries to the abdomen, legs, or back. Height and weight were measured by a physiotherapist before the start of the study. For the other inclusion and exclusion criteria, the participants completed an eligibility questionnaire. The L-DOMS group was matched one-to-one with L-PAIN based on age (± 5 years), sex, and BMI (± 3 kg/m^2^).

### Eccentric exercise protocol (L-DOMS)

**Fig. 1:**
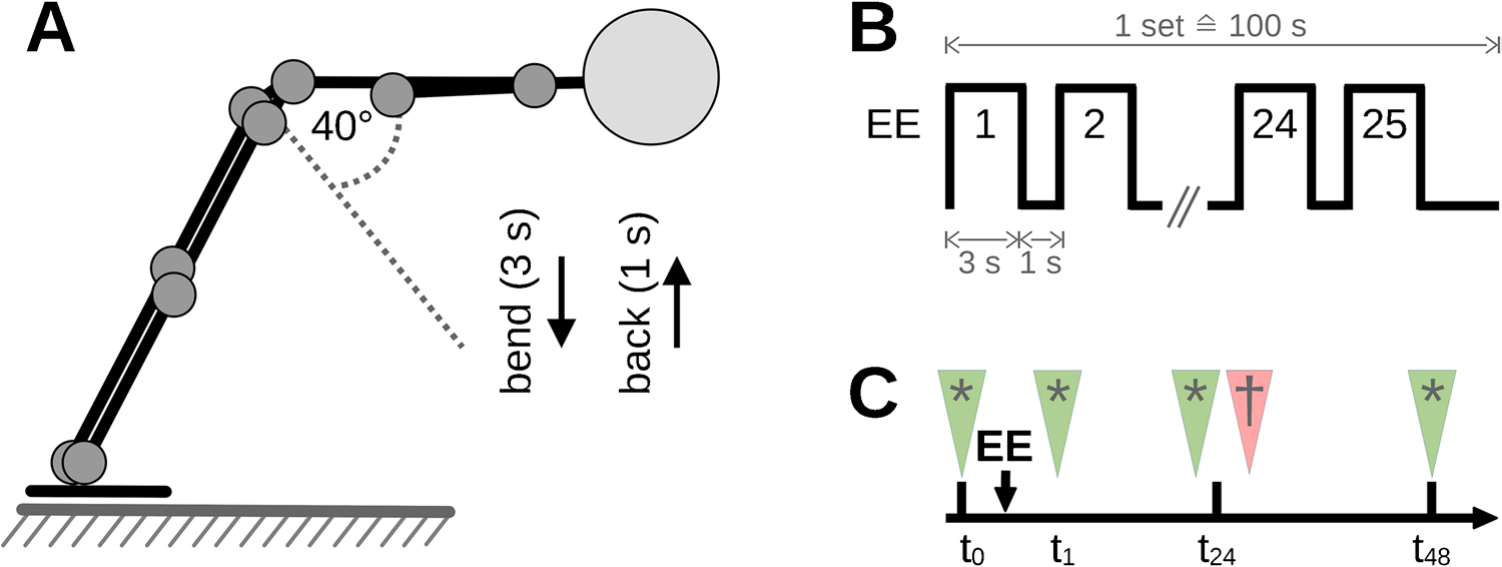
**A** Schematic drawing of the eccentric fatigue protocol to induce DOMS. **B** Protocol for eccentric exercise. **C** Experimental schedule. EE, eccentric exercise; s, second; *t*_0_, baseline; *t*_1_, post-exercise; *t*_24_, 24 h after exercise; *t*_48_, 48 h after exercise; * (green arrows), pressure pain threshold, self-reported delayed onset muscle soreness; † (red arrow), Pain Perception Scale survey

To induce DOMS in the present study, using a back extension bench (Finnlo Tricon, Hammer Sport AG, Neu-Ulm, Germany), L-DOMS participants bent their trunk from the starting position parallel to the floor into a 40° flexion position for 3 s and then returned it to the starting position as quickly (ca. 1 s) as possible (Fig. 1A). One set consisted of 25 repetitions with a rest period in flexed position of 10 s (Fig. 1B). Sets were repeated under time announcement of the examiner (certified fitness trainer and sports scientist with more than 10 years of experience instructing exercises) until the participants were subjectively exhausted and could no longer continue the exercise.

This eccentric exercise protocol was selected because it is commonly used in pain research to induce long-lasting experimental aLBP through back muscle fatigue [3, 10, 31, 35, 55]. Bishop et al. [3] showed a pain increase of 16 mm (standard deviation; SD = 2 mm) and 15 mm (SD = 2.5 mm) on the visual analogue scale (VAS) at 24 and 48 h post exhausting eccentric trunk extensions. Pain decreased by 11 mm (SD = 1 mm) after 96 h. Dannecker et al. [16] found a 24-h increase of 17.3 mm (SD = 2.15 mm) and a 48-h increase of 21.3 mm (SD = 2.38 mm) using a similar protocol in men. Therefore, eccentric exercise-induced DOMS can be concluded to produce clinically relevant but temporally limited pain peaking at 24 to 48 h [3, 16, 17, 47].

### Outcomes for delayed onset muscle soreness group (L-DOMS)

In the L-DOMS group, self-reported DOMS and PPT were measured before (*t*_0_), immediately after (*t*_1_), one day (*t*_24_), and 2 days (*t*_48_) after the exercise protocol. However, due to software problems, the measurement data for the PPT were not available for *t*_1_. In addition, qualitative pain scores were obtained at time *t*_24_, as it was described that induced pain peaks 24 h after eccentric loadings pain qualities were inquired at time *t*_24_ [42] (Fig. 1 C).

#### Self-reported DOMS

The method of Lau et al. [32] was used to quantify DOMS. Here, an investigator palpated the multifidus muscle at the L3/L4 level, 40 mm lateral to the spinous process, in longitudinal direction and applied a pressure of about 400 kPa with the tips of the middle and index fingers of the right hand, which was repeated for three times. The palpation point was marked with adhesive tape for reference. A 100-mm VAS was used according to Lau et al. [32] to ask participants to indicate the level of pressure pain. Thereby, 0 indicates no pain and 100 indicates most imaginable pain. The experimenter was trained with a force gauge prior to data collection to ensure that the correct pressure was applied with at most 5% variation between trials [32]. VAS-based measurement of palpation pain at the biceps brachii muscle showed a high reliability of ICC ranging from 0.98 to 0.99 [40].

#### Pressure pain threshold

The PPT of the multifidus muscle was measured at the palpation point using a digital algometer (IndentoPro, Fascia Research Group, University of Ulm; Institute of Human Movement Sciences, University of Chemnitz, Germany). The 100-mm^2^ probe was placed perpendicularly on the muscle of the subject in prone position, and the pressure force was gradually increased at 50 kPa/s until the subject felt the first sensation of pain (stinging, pricking, or burning sensation). The measurement was then repeated twice after a 10-s rest period, and the average of the measurements was used for further analysis. This procedure was described as very reliable with an ICC of 0.92 to 0.98 [12].

#### Pain perception scale

DOMS pain quality was assessed with the SES [22]. The scale consisted of a validated list of 14 affective and 10 sensory descriptors rated on a four-level ordinal scale (0, no match; 1, light match; 2, largely match; 3, total match).

### Pain perception scale outcomes for L-PAIN group

To compare the pain induced by the DOMS protocol in this study and established myofascial pain patterns, data from a previous study [51] were secondary analyzed and included. Briefly, the comparison (L-PAIN group) consisted of SES outcomes from *n* = 16 participants which were assessed for muscle- and fascia-excited primary nociceptive afferents. Participants were electrically stimulated (single stimuli at twice the magnitude of the individual pain threshold and trains of high-frequency stimuli, 100 Hz for 1 s, at 10 times the individual electrical detection threshold were used to elicit pain) with concentric bipolar needle electrodes inserted into (a) the multifidus muscle and (b) the thoracolumbar fascia under ultrasound guidance. The test order was crossover balanced for right-left and tissue type. Subsequent to tissue stimulation, pain qualities for both muscle and fascia stimulation were assessed with the SES. For the full procedure, see Schilder et al. [51].

### Statistical analysis

Mean, SD, and 95% confidence interval (95% CI) were determined for the continuous outcomes.

Factor analysis of sensory descriptors of the SES accepting factors with eigenvalues > 1 was used to reduce the complexity of sensory patterns. Subsequent factor rotation using normalized VARIMAX yielded orthogonal factors with maximal factor separation.

The resulting factors were *z*-score normalized to the grand mean and SD of the respective data origins. Subsequently, an one-way ANOVA was performed to the compare L-DOMS and L-PAIN myofascial sensory pain descriptors collapsed by factor analysis. Significant results were examined post hoc using Tukey’s HSD test.

Coefficients of variation (CV) and their 95% CIs for the sensory pain descriptors were calculated to test for possible differences in the dispersion of pain perception between the groups. Following our hypothesis that DOMS relates more to fascial pain, the absolute differences between matched pairs of sensory fascia pain and DOMS descriptors and SD were calculated.

Repeated measures ANOVA and Tukey’s HSD were performed for PPT and self-reported DOMS. ANOVA partial *η*^2^ effect sizes were interpreted according to Cohen for > 0.01 as small, > 0.06 as medium, and > 0.14 as large [14]. All outcomes met the assumptions for parametric testing (*p* > 0.05). Analyses were performed using the Jamovi 2.3 (The jamovi project, https://www.jamovi.org).

## Results

**Fig. 2:**
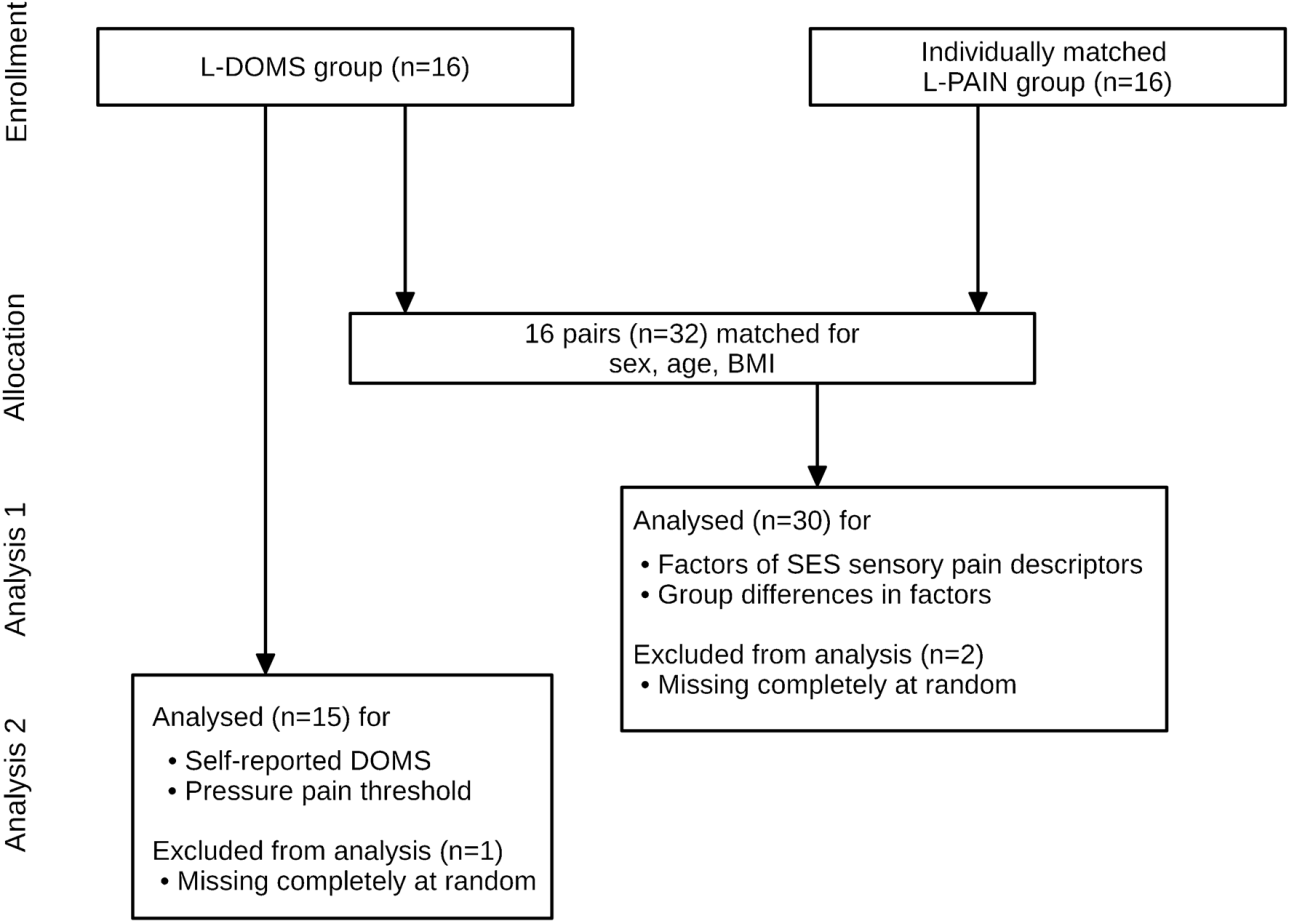
Flow diagram of the study. L-DOMS, delayed onset muscle soreness in the lower back; L-PAIN, data analysis by Schilder et al. [51]; *n*, number; BMI, body mass index

Participants were successfully matched for age, sex, and BMI. The study was conducted from 04/27/2023 to 04/29/2023. No adverse events were recorded, and baseline data were not different between groups according to a Student’s *t*-test (Table 1). One participant in the L-DOMS group was prevented from attending the study (missing completely at random). Therefore, the matched pair was excluded listwise from the analysis (Fig. 2).

**Table 1:** Baseline characteristics

			95% confidence interval			
	Group	Mean	Lower	Upper	SD	*p*-value
Sex (w/m)	L-DOMS	7/8				
	L-PAIN	7/8				
Age (years)	L-DOMS	25.89	23.27	28.52	4.73	
	L-PAIN	24.00	22.91	25.09	1.96	0.164
Height (m)	L-DOMS	1.74	1.66	1.81	0.12	
	L-PAIN	1.75	1.69	1.80	0.09	0.788
Weight (kg)	L-DOMS	70.93	63.08	78.78	14.17	
	L-PAIN	67.20	60.23	74.17	12.58	0.452
BMI (kg/m^2^)	L-DOMS	23.11	21.60	24.63	2.78	
	L-PAIN	22.26	21.11	23.41	2.07	0.344

*SD*, standard deviation; *BMI*, body mass index

**Fig. 3:**
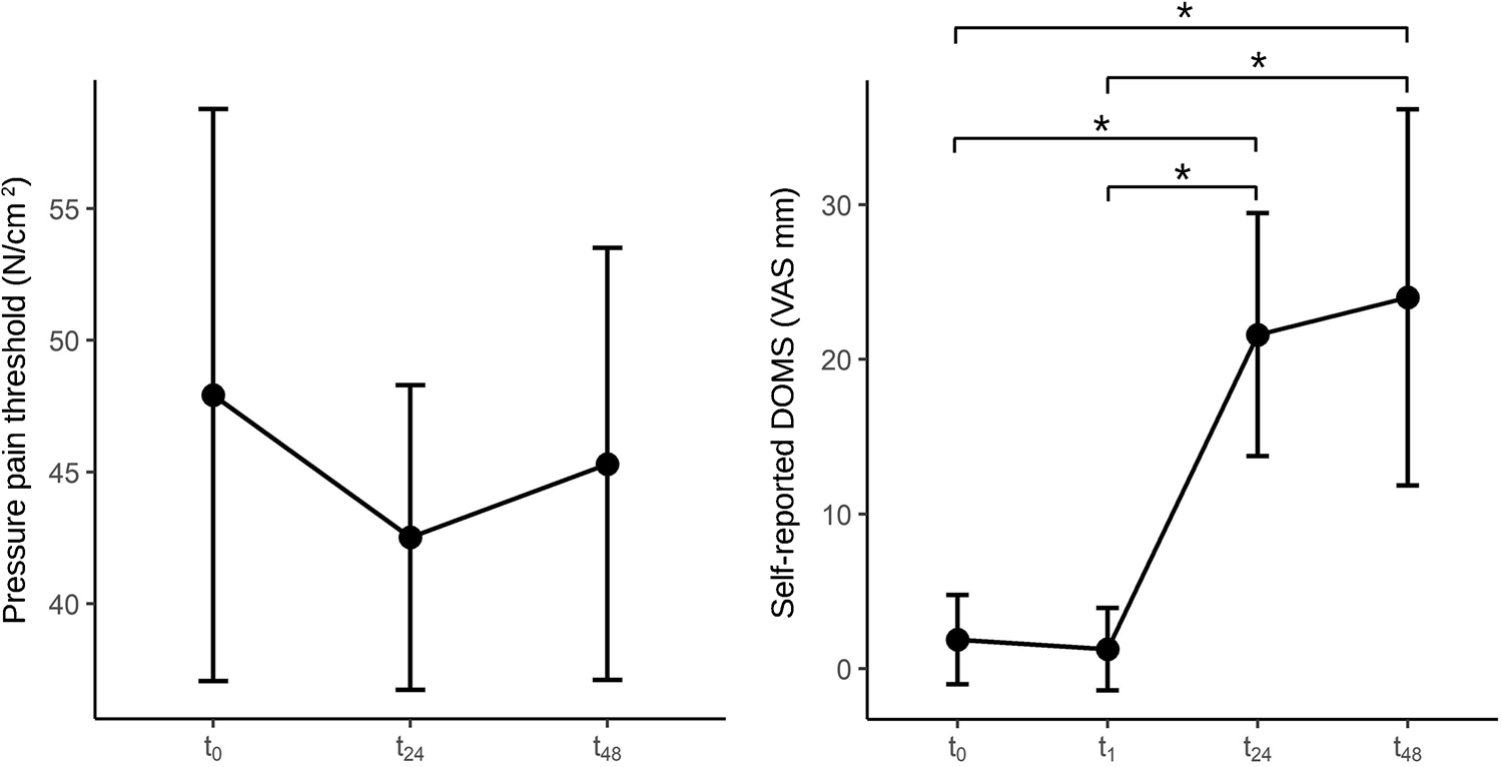
Continuous outcomes. *t*_0_, pre-measurement; *t*_1_, measurement after eccentric exercise; *t*_24_, measurement 24 h after eccentric exercise; *t*_48_, measurement 48 h after eccentric exercise; VAS, Visual Analogue Scale. Error bars represent the 95% confidence interval. *Significant at *p* < 0.05

Repeated-measures ANOVA revealed no differences for PPT, *F*(2, 28) = 0.269, *p* = 0.766, partial *η*2 = 0.019. There was a significant difference for self-reported DOMS, *F*(3, 42) = 14.0, *p* < 0.001, partial *η*2 = 0.499. Tukey’s post hoc comparisons showed significant differences between *t*_0_ and *t*_24_ (-19.60 mm; *p* = 0.003), between *t*_0_ to *t*_48_ (− 22.00 mm; *p* = 0.006), between *t*_1_ and *t*_24_ (− 20.27 mm; *p* = 0.002), between *t*_1_ to *t*_48_ (− 22.67 mm; *p* = 0.004), but not between *t*_0_ and *t*_1_ (0.67 mm; *p* = 0.752), and not between *t*_24_ and *t*_48_ (− 2.40 mm; *p* = 0.973). Descriptive statistics are shown in Table 2 and Fig. 3.

**Table 2:** Descriptive statistics of continuous outcomes

			95% Confidence Interval		
		Mean	Lower	Upper	SD
PPT (N/cm^2^)	*t*_0_	45.68	34.04	57.32	21.01
	*t*_24_	42.95	36.26	49.65	12.08
	*t*_48_	43.58	34.83	52.34	15.81
Pain (VAS mm)	*t*_0_	1.88	− 1.02	4.77	5.44
	*t*_1_	1.25	− 1.41	3.91	5.00
	*t*_24_	21.60*	13.74	29.46	14.18
	*t*_48_	24.00*	11.83	36.17	21.97

PPT, pressure pain threshold; Pain, self-reported DOMS. *t*_0_, pre-measurement; *t*_1_, measurement after eccentric exercise; *t*_24_, measurement 24 h after eccentric exercise; *t*_48_, measurement 48 h after eccentric exercise; VAS, Visual Analogue Scale

*Significant to *t*_0_ and *t*_1_ at *p* < 0.05

Ratings of affective items of the SES were generally very low and did not differ from 0 in 7 of 14 participants in the L-DOMS group (mean ± SD: 0.098 ± 0.131; L-PAIN group mean ± SD: 0.639 ± 0.731). Therefore, sensory pain descriptors for L-DOMS and L-PAIN SES were further used and reduced by factor analysis, and factor separation was maximized by VARIMAX rotation, yielding three orthogonal sensory factors that explained 77.8% of the variance, namely, “heat pain” (high factor loadings on the items “scalding” and “hot”), superficial sharp pain (high loadings on “cutting,” “tearing,” and “stinging”), and deep pain (high loadings on “beating,” “throbbing,” and “pounding”). The item “burning” loaded jointly on the factors “sharp pain “ and “heat pain,” and the item “piercing” loaded on “sharp pain “ and “deep pain” (Table 3).

**Table 3:** Sensory factor loadings after normalized VARIMAX rotation

	Sharp pain	Deep pain	Heat pain	Uniqueness
Cutting	0.834			0.281
Beating		0.835		0.239
Burning	*0.565*		*0.617*	0.222
Tearing	0.779			0.309
Throbbing		0.940		0.115
Scalding			0.853	0.199
Stinging	0.853			0.232
Pounding		0.899		0.186
Hot			0.919	0.143
Piercing	*0.640*	*0.546*		0.292
Variance explained (single factor)	28.6%	28.3%	20.9%	
Variance explained (total)		77.8%		

Loadings below 0.3 are not shown

**Fig. 4:**
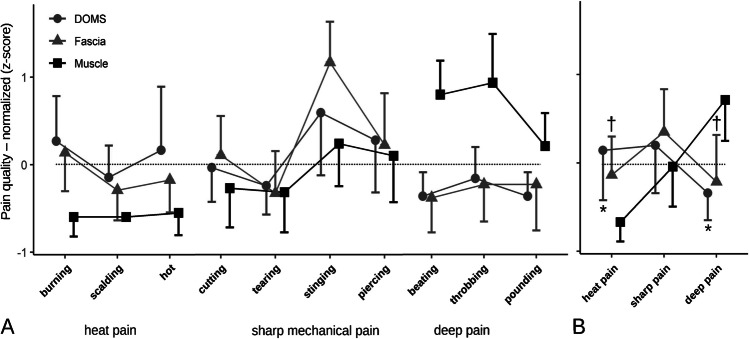
Pain qualities of DOMS, fascia, and muscle stimulation. **A** Sensory descriptors were ordered according to a 3-factor model determined independently for DOMS (this study) and fascia/muscle (assorted data analysis by Schilder et al. [51]). **B** Factor analysis collapsed items. Data were normalized to the overall mean and SD of each data origin and tissue. *Significant difference between DOMS and muscle at *p* < 0.05 level; †Significant difference between fascia and muscle at *p* < 0.05 level

There were two outliers in the “heat pain” factor dataset which were replaced by 95 percentile Winsorizing. One-way ANOVA revealed significant differences regarding the “heat pain” factor between DOMS, muscle and fascia, *F* (2, 28) = 5.48; *p* = 0.010; partial *η*^2^ = 0.28. Tukey’s HSD showed significant differences between DOMS and muscle (0.920; *p* = 0.027) and fascia and muscle (0.655; *p* = 0.029), but not between DOMS and fascia (0.266; *p* = 0.679). One-way ANOVA revealed no significant differences regarding the “sharp pain” factor between DOMS, muscle and fascia, *F* (2, 28) = 0.933; *p* = 0.405; partial *η*^2^ = 0.062. One-way ANOVA revealed significant differences regarding the “deep pain” factor between DOMS, fascia, and muscle, *F* (2, 28) = 9.08; *p* < 0.001; partial *η*^2^ = 0.393. Tukey’s HSD showed significant differences between DOMS and muscle (− 1.125; *p* = 0.002) and between fascia and muscle (− 1.076; *p* = 0.001), but not between DOMS and fascia (− 0.049; *p* = 0.990; Table 4; Fig. 4).

**Table 4:** Descriptive statistics of collapsed pain perception items

				95% confidence Interval		
	Group	*N*	Mean	Lower	Upper	SD
Heat pain	DOMS	16	0.143*	− 0.473	0.759	1.16
	Fascia	16	− 0.062†	− 0.491	0.367	0.81
	Muscle	16	− 0.696	− 0.987	− 0.404	0.55
Sharp pain	DOMS	16	0.198	− 0.389	0.785	1.10
	Fascia	16	0.351	− 0.176	0.877	0.99
	Muscle	16	− 0.042	− 0.536	0.451	0.93
Deep pain	DOMS	16	− 0.341*	− 0.676	− 0.005	0.63
	Fascia	16	− 0.214†	− 0.789	0.362	1.08
	Muscle	16	0.679	0.203	1.154	0.89

*Significant difference between DOMS and muscle at *p* < 0.05 level

†Significant difference between fascia and muscle at *p* < 0.05 level

The CV for the sensory pain descriptors of DOMS was 0.45 (95% CI = 0.38–0.53), for fascia pain 0.42 (95% CI = 0.35–0.50), and for muscle pain 0.41 (95% CI = 0.34–0.48). The mean difference between pain descriptors of fascia pain and DOMS was 0.48 (SD = 0.94), with the exception of three pairs (two had a difference of 3, one of 2.5) where the difference was less than or equal to 2.

## Discussion

There is a considerable density of nociceptive afferents in osseous, muscle, and deep fascial tissue [6]. DOMS has been hypothesized to elicit muscle-related pain and is therefore used as a surrogate for aLBP [3, 31, 35, 55]; however, to date, an investigation of pain quality after exercise-induced DOMS has been lacking. To our knowledge, the present study is the first to analyze pain-related outcomes and compare them with muscle and fascial pain. We found that pain descriptors for DOMS and fascia pain overlapped in a striking manner, while DOMS and muscle pain differed significantly, suggesting fascial rather than a muscular pain origin.

### Pressure and palpation pain

There were no differences in PPT after induction of DOMS compared with baseline, whereas palpation pain increased significantly 24 h and 48 h after eccentric exercise. These results are consistent with those of Tenberg et al. [57], who also found no increase in PPT in DOMS participants, but an increase in palpation pain. It is well known that hyperalgesia to blunt pressure (such as the indentation of 1 cm in this study) is elicited only to a small extent by superficial afferents and that peripheral sensitization of nociceptive afferents from deeper tissues are the primary mechanism here [25, 29].

Injections of hypertonic saline into the muscle are capable of eliciting significant PPT changes, but injections into the overlying fascia are not [49]. It is therefore suggested that the PPT is more likely to affect muscle nociceptors, which, however, were probably not stimulated in this and the aforementioned study.

An increase in DOMS after eccentric exercise has traditionally been related to skeletal muscle, such as structural sarcoma damage to the Z-disc [21], excessive lactate production [24], or free radical accumulation [13]. Tenberg et al. [57] found swelling of the ECT in DOMS and a strong correlation with reported pain. Wilke et al. [65] further found stiffening of the ECT in DOMS, which also correlated with pain, but no stiffening of the muscle. It is therefore hypothesized that the increase in palpation pain in this study may have resulted from greater stimulation of nociceptors in the ECT due to longitudinal movement with constant pressure of sufficient magnitude by the examiner.

### Sensory pain description patterns

DOMS pain descriptions were statistically equivalent to those after electrically induced pain in the TLF, showing higher scores for “heat pain” as well as “sharp pain” and generally lower scores for “deep pain.” Traditionally, “heat pain,” separated by the factors “hot” and “burning,” which were among the most frequently selected descriptors, has been attributed to C-fiber-mediated second pain [28] and is also considered a prototypical neuropathic pain [2]. However, for both fascia pain and DOMS, the “sharp pain” descriptors, “piercing,” and “stinging,” which are mostly attributed to the A-delta-mediated first pain, were also commonly selected [2]. Nevertheless, more recent studies found that the descriptors “stinging” in combination with “burning” were also used to characterize selective A-delta fiber stimulation [2, 33]. It is very likely that, given the failure to meet diagnostic criteria, DOMS and electrically stimulated deep tissue pain do not have a neuropathic origin, raising the question of whether sometimes low back pain is also misinterpreted as neuropathic [19].

In contrast, electrical stimulation of the multifidus muscle (L-PAIN group) was followed by classic “deep pain” and differed significantly in this quality from DOMS (L-DOMS group) and fascia pain (L-PAIN group), supporting the distinct differences between previous claims about the muscle-related origin of DOMS and the study results [3, 31, 35, 55]. These typical, significantly different fascial and muscle pain descriptors were also observed after hypertonic saline injection, demonstrating here that chemical and electrical stimulation produce similar results [49]. It is notable that the factor analysis applied to the SES in this study yielded an almost identical three-factor structure to two studies that previously examined fascia and muscle with the SES [50, 51]. Although both the multifidus muscle and the adjacent ECT refer to the deep tissues of the lumbar region, the quality pattern of “deep pain” was identified only in the muscle, whereas the pain pattern of the fascia corresponded more to the DOMS of “heat pain” and “sharp pain,” which also tended to be attributed to the superficial tissues [51].

### Implications for researchers

Together with recent findings, the study results do not provide evidence that DOMS is a surrogate of muscle pain [32, 57, 62, 65]. However, this is not inconsistent with DOMS itself being a model that can endogenously produce clinical spontaneous pain similar to that seen in aLBP [3, 51, 54, 62, 64]. The TLF has been discussed as a possible source of low back pain [54, 64]. Langevin et al. [30] demonstrated morphological changes in the TLF in patients with chronic low back pain leading to a reduction in shear strain. Brandl et al. [7] showed that these mechanisms are also present in aLBP patients and probably alter muscle activity. Therefore, several mechanisms for TLF-mediated low back pain have been discussed. First, nociceptive free nerve endings could be directly irritated by microinjuries [64]. Second, morphologic alteration following these microinjuries may impair proprioceptive signaling and/or trigger hypoxia-induced inflammation [8]. This could lower the pain threshold through fascia-dependent sensitization of large dynamic range neurons [48]. Researchers using DOMS as a clinical model for aLBP should be aware of these mechanisms and avoid falling back on older concepts that muscle pain would be exclusively induced here. They should also consider the likely contribution of superficial tissue, ECT, in particular TLF, to the development of DOMS and also aLBP.

### Limitations

Both stimulation of the TLF with bipolar needle electrodes and DOMS result in relatively low levels of pain compared with previously observed skin stimulation and could result in lower spatial summation of pain [49]. Since fascia pain and muscle pain are dependent on the stimulation intensity [59], the level of DOMS-induced pain might affect experimentally evoked pain qualities in another cohort. However, the mean values were very similar to the pooled mean value from a systematic review of aLBP, indicating that the methods used were capable of producing a similar level of pain as true aLBP [44]. Furthermore, the present study investigated the pain perception of DOMS in the low back area. Since fascia afferents from different body areas are known to show different levels of somatosensory effects after stimulation [34], the site of DOMS might also influence the perception of pain.

Pain perception varies between individuals [41]. In this study, the data of different participants were analyzed secondarily by matching the group members in terms of age, sex and BMI. However, this does not take into account the potentially different pain perception of both groups. Since invasively inducing pain is questionable from an ethical [27] and methodological [45] point of view just to find suitable comparison matched samples, we however decided to accept this shortcoming and compare the sensory pain descriptors in terms of CV and absolute differences. The results showed only marginal variation in this respect, indicating that the two study populations were comparable.

Participants were young and healthy, as required by the inclusion criteria and because of comparability, but this is a common problem when using pain models and, per se, although necessary to investigate the main mechanisms, may not reflect real patients with aLBP [36].

## Conclusion

Electrical stimulation of fascia and exercise-induced DOMS result in similar pain description patterns, whereas descriptions of DOMS and muscle pain differ significantly. This finding suggests that DOMS pain may rather be of fascial than of muscular origin.

### Supplementary Information

Below is the link to the electronic supplementary material.

Supplementary file1 (XLSX 11 KB)

## Data Availability

The raw data supporting the conclusions of this article will be made available by the authors, without undue reservation.
